# Spatial distribution of tertiary lymphoid structures in the molecular and clinical context of non-small cell lung cancer

**DOI:** 10.1007/s13402-025-01052-x

**Published:** 2025-03-03

**Authors:** Hedvig Elfving, Hui Yu, Kaleab Kassete Fessehatsion, Hans Brunnström, Johan Botling, Miklos Gulyas, Max Backman, Amanda Lindberg, Carina Strell, Patrick Micke

**Affiliations:** 1 Department of Immunology, Genetics, and Pathology, Uppsala University, Uppsala, 751 85 Sweden; 2 Division of Pathology, Department of Clinical Sciences Lund, Lund University, Lund, Sweden; 3 Centre for Cancer Biomarkers CCBIO, Department of Clinical Medicine, University of Bergen, Bergen, Norway

**Keywords:** Immunotherapy, Tumor microenvironment, Prognosis, Lymphocytes, Immune microenvironment

## Abstract

**Introduction:**

Tertiary lymphoid structures (TLS) are lymphocyte aggregates resembling secondary lymphoid organs and are pivotal in cancer immunity. The ambiguous morphological definition of TLS makes it challenging to ascertain their clinical impact on patient survival and response to immunotherapy.

**Objectives:**

This study aimed to characterize TLS in hematoxylin-eosin tissue sections from lung cancer patients, assessing their occurrence in relation to the local immune environment, mutational background, and patient outcome.

**Methods:**

Two pathologists evaluated one whole tissue section from resection specimens of 680 NSCLC patients. TLS were spatially quantified within the tumor area or periphery and further categorized based on the presence of germinal centers (mature TLS). Metrics were integrated with immune cell counts, genomic and transcriptomic data, and correlated with clinical parameters.

**Results:**

TLS were present in 86% of 536 evaluable cases, predominantly in the tumor periphery, with a median of eight TLS per case. Mature TLS were found in 24% of cases. TLS presence correlated positively with increased plasma cell (CD138+) and lymphocytic cell (CD3+, CD8+, FOXP3+) infiltration. Tumors with higher tumor mutational burden exhibited higher numbers of peripheral TLS. The overall TLS quantity was independently associated with improved patient survival, irrespective of TLS maturation status. This prognostic association held true for peripheral TLS but not for tumor TLS.

**Conclusion:**

TLS in NSCLC is common and their correlation with a specific immune phenotype suggests biological relevance in the local immune reaction. The prognostic significance of this scoring system on routine hematoxylin-eosin sections has the potential to augment diagnostic algorithms for NSCLC patients.

**Supplementary Information:**

The online version contains supplementary material available at 10.1007/s13402-025-01052-x.

## Introduction

Immune checkpoint inhibitors are now implemented in the treatment algorithm of many cancer types and can provide long-term survival in subsets of advanced cancer patients [1]. The clinical success in a minority of patients suggests the relevance of the tumor immune environment and that specific anti-cancer immune reactions can be activated. There is also experimental and clinical evidence of the immune system’s influence in the natural course of cancer, by inhibiting or stimulating tumorigenesis. Indeed, immune cells in or around the tumor compartment imply strong prognostic information [2–6].

In non-small cell lung cancer (NSCLC), the in situ densities of immune cells and their subsets demonstrate an association between high densities of infiltrating T-lymphocytes and B-cells with long-term survival [5, 7–11]. Plasma cells, in lung cancer and many other cancer types, have a particularly strong association with longer survival [12–15]. Conversely, when inhibitory immune cells, like regulatory T-cells or M2-like macrophages, are abundant in the local tumor environment the prognosis for these NSCLC patients is dismal [16, 17]. Furthermore, with the introduction of stable multiplex techniques, combined with advanced image analyses, it is possible to locally define the spatial relations of immune cells to tumor cells or immune cell subsets (CD8 + effectors cells or CD4 + memory T-cells) to each other [3, 7, 8, 18, 19]. These studies demonstrate that the spatial context and cellular interactions are clinically important and provide strong prognostic or predictive information [20–22].

One striking immune pattern recognized long ago by pathologists, in standard hematoxylin-eosin (H&E) histological sections from diagnostic cancer samples, are aggregates of lymphocytes gathered in rounded, organized structures with or without germinal centers, resembling the architecture seen in secondary lymphoid organs such as lymph nodes. Although a very common phenomenon in cancer tissue and a variety of inflammatory conditions, these aggregates were at a later stage specified and termed tertiary lymphoid structures (TLS) [23–26] and described in all solid cancer types, including NSCLC [27–29].

Recently, the cellular composition of TLS was characterized in detail using immunohistochemical markers on NSCLC tissue, and have been found to consist of B-cells (CD20 + or CD19+), follicular dendritic cells (CD21+, CD35+, and CD23+), plasma cells (CD138 + and CD269+), T-cells (CD3+,CD8+, or CD4 + subpopulations), T-helper 1 cells (intracellular T-bet+), regulatory T-cells (FOXP3+), dendritic cells (DC-LAMP+, CD83+, or CD86+), and macrophages (CD68+). Furthermore, high endothelial venules (PNAd + and MECA79+), typical vascular structures of lymph nodes, were found to be associated with TLS [27, 30–33].

Indeed, as for plasma cells, the presence of TLS in NSCLC is consistently associated with a favorable prognosis [25, 27, 31, 32]. Maybe of greater clinical impact, TLS have been shown to be predictive for the response of immunotherapy in different cancer types as well as in NSCLC [34, 35].

Taken together, TLS are suggested to be of biological and clinical relevance in NSCLC. However, most previous studies were relatively small and their annotations have been based on various methods, mostly immunohistochemical staining with manual or automated annotations [27, 31, 32, 36–39]. In addition, the relation between localized lymphocyte aggregates and the overall immune repertoire of the cancer environment was incompletely addressed. With this work, we aim to provide a more comprehensive outline of the presence of TLS, with an annotation method that can be performed on the existing diagnostic sections by a pathologist. The occurrence and distribution of TLS are integrated into an extensively clinically and molecularly annotated NSCLC cohort.

## Materials and methods

### Patient cohort

The source study population consisted of two previously described cohorts of, in total 680 NSCLC patients, surgically treated at the Uppsala University Hospital (Suppl. Table 1). The first cohort (Uppsala I), consisting of 354 patients, included patients who underwent surgical tumor resection between 1995 and 2005 [40]; the second cohort (Uppsala II), consisting of 326 patients, included patients who underwent surgical resection between 2006 and 2010 [41]. The follow-up was at least 5.0 years (median 9.4 years) for Uppsala 1 and 8.0 years (median 10.2 years) for the Uppsala II cohort. Patients receiving neoadjuvant chemotherapy were excluded from the cohorts.

### Histopathological evaluation

One representative H&E-stained tumor slide including tumor periphery and adjacent non-tumorous tissue were selected and was scanned in 40x on a Hamamatsu NanoZoomer S60 (Hamamatsu photonics k.k., Japan) and assessed using the Hamamatsu NDP.view2 viewer (www.hamamatus.com/jp/en.html). The size of the selected tumor sections were relatively comparable, based on the standard blocs used in the diagnostic routine measuring approximately 16–22 mm in length and 10–12 mm in width. Slides with inadequate tissue quality, extensive necrosis, too little area of tumor tissue, or without assessable tumor border area were excluded.

**Fig. 1:**
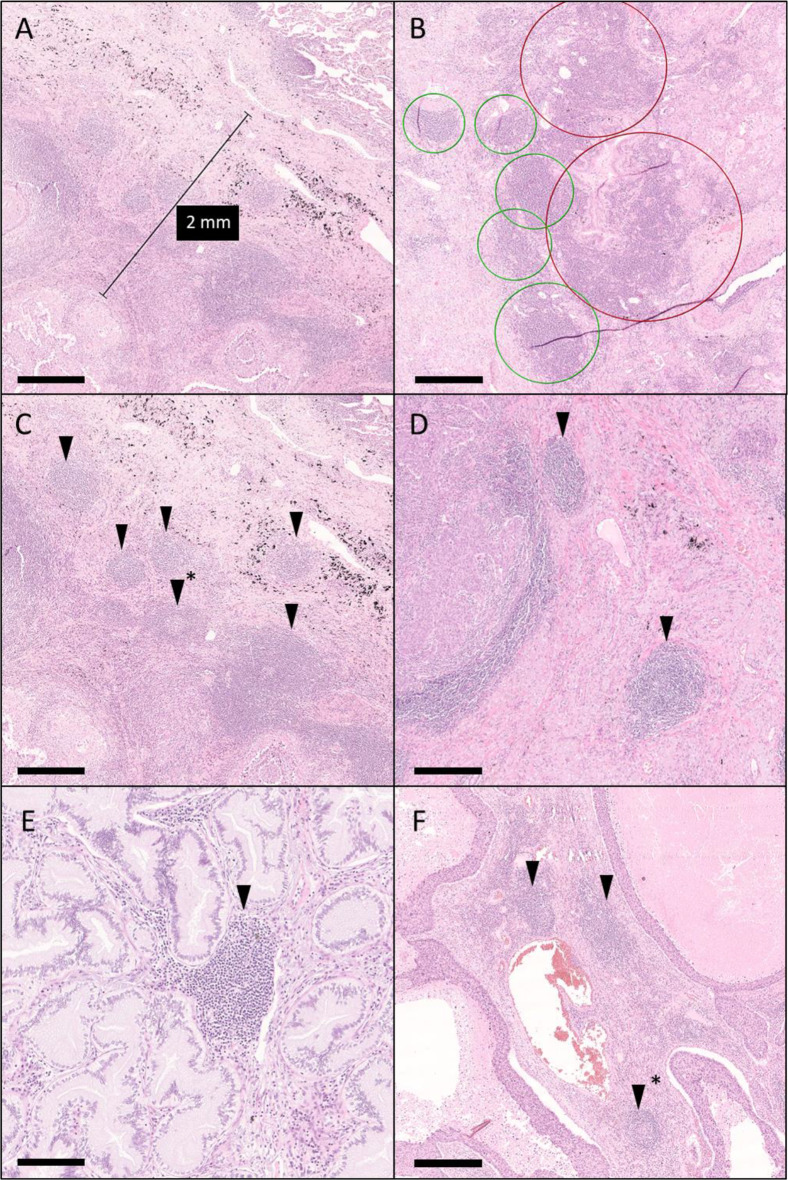
Histological images exemplifying the annotation of TLS in NSCLC. **A**: The evaluation of TLS was done separately in the tumor area and the tumor periphery. The periphery was defined as the region within 2 mm distance from the nearest tumor border (black line). **B**: TLS were defined as circumscribed lymphoid aggregates (green circles). Diffuse areas of dense inflammation were not regarded as TLS (red circle). **C**-**F**: Examples of TLS in the tumor periphery (**C**-**D**) and in the tumor area (**E**-**F**) (* indicate germinal center). Scale bar indicate 500 μm

The distribution of TLS was annotated in the tumor area or periphery according to the study of Rakaee and colleagues with minor modifications [39]. TLS were considered intratumoral if located within the tumors’ margin. The periphery was defined as the region including non-malignant tissue within 2 mm from the tumor invasive margin. A TLS was defined as a dense, nodular, aggregate of lymphoid cells resembling a lymphoid structure with or without germinal center, with a minimum size of 150 μm. If the TLS displayed a central area of lighter cells, indicating the formation of a germinal center, they were annotated as mature TLS. Fused TLS with multiple germinal centers were uncommon, but when encountered were counted as separate TLS. Areas with strong diffused lymphocytic infiltration, without recognizable lymphoid follicles/aggregates, were not included in the analysis. Lymphocytic aggregates were not counted if residing in lymph nodes and normal alveolar structures. One specialist pathologist (HE) and one resident pathologist (KFF) independently evaluated the whole cohort and performed a manual, compartment-specific count of TLS numbers. An independent pulmonary pathologist (PM) reevaluated deviating cases. The final statistical analysis was based on the specialist’s annotation. Histological photos exemplifying TLS, and their evaluation are presented in Fig. 1.

### Mutation data

Genomic analysis was performed previously on subsets of patients. This included *EGFR* (*Sanger sequencing*), *TP53* (*Sanger sequencing*), and *KRAS* mutations (*pyrosequencing*) for 457 patients [42, 43] as well as targeted sequencing for 352 patients including 82 lung cancer-related genes and an estimation of tumor mutational burden (TMB) [44]. Targeted sequencing was performed using the Haloplex target enrichment system (Agilent Technologies, Santa Clara, CA, USA) with 200 ng input DNA. An assay targeting the coding exons of 82 genes was used as previously described [44]. The design covers a total region of approximately 0.47 Mb and uses 39,328 probes to capture the targeted genes. The enriched and amplified samples concentrations were determined using D1000 Screen Tapes (Agilent). Subsequently the samples were pooled in equimolar amounts and thereafter purified using the Agencourt AMPure XP system (Beckman Coulter, Indianapolis, IN, USA). The pooled samples were then sequenced on the Illumina HiSeq 2500 platform.

The *ALK* status was assessed by immunohistochemistry (Ventana Ventana ALK (D5F3) CDx Assay) as previously described [45] and eventually confirmed by FISH analysis (Vysis ALK Break Apart FISH Probe Kit).

### Quantification of immune cell infiltrates

Information on immune cell infiltrates, annotated on tissue microarrays (TMAs) in the form of immune cell scores, was available for 271 patients and was previously described [14, 46]. In brief, TMAs were constructed containing two 1 mm cores from areas best representing the central tumor areas. The TMAs were stained for lymphocytic markers (CD3, CD4, CD8, and FOXP3), M2 like macrophages/myeloid cells (CD163), plasma cells (CD138), and the checkpoint molecules PD-1 and PD-L1. The TMAs were then visually annotated for immune marker-positive cells in the respective tumor and stroma compartment. The immune cell score was calculated by dividing the number of stained cells in a compartment with all viable cells in that compartment.

### Statistical analysis

Standard descriptive statistics were used to present the clinical characteristics of the cohort. Intraclass correlation was used to determine the interrater reliability, i.e. to compare the TLS counts between the two pathologists. The level of agreement was rated accordingly: [47] 0 = agreement equivalent to chance; 0.10–0.20 = slight agreement; 0.21–0.40 = fair agreement; 0.41–0.60 = moderate agreement; 0.61–0.80 = substantial agreement; 0.81–0.99 = near‑perfect agreement; 1.00 = perfect agreement.

The TLS annotations were dichotomized using the minimum *P*-value method [31, 48] as follows: all TLS/peripheral TLS 0–7, vs. >7; mature TLS 0–2 vs. >2. The relationship between number of TLS and clinical parameters was assessed with Wilcoxon-Mann-Whitney. Immune cell infiltrates and TMB were correlated to TLS with the Spearman rank correlation. For correlation with tumor mutation status, Wilcoxon-Mann-Whitney test was used. The survival analyses were assessed with the Kaplan-Meier method and the difference between the groups was compared with the log-rank test. Univariate and multivariate Cox regression models were used to evaluate the relative risk of death. The multivariate model was adjusted for established clinical parameters: age, stage, smoking, and performance status. The clinical parameters were dichotomized as follows; age < 70 vs. ≥70 years; tumor stage I vs. II-IV; smoking ever smoker vs. never smoker; performance status 0 vs. 1–4. Adjustment for multiple testing was done by the Benjamini-Hochberg method. *P* <.05 indicated statistical significance. All statistical analysis was performed in R Studio (version 2023.06.0, build 421) including packages irr, survminer, survival, and ggsurvplot.

## Results

### Histopathological evaluation

The diagnostic H&E slides of the complete source cohorts of 680 cases were reviewed for slides containing representative tumors with a non-cancerous border area. Finally, 536 cases were selected and one representative slide per case was included in the subsequent evaluation (Table 1: characteristics of the study cohort; Suppl. Table 1: comparison between the study cohort and the source cohorts). The clinicopathological characteristics of the source cohorts and the study cohort did not show significant differences *(P* >.35, all comparisons). The number of TLS, with and without germinal centers, were manually counted and annotated by a trained pathologist (HE) and a resident (KKF) separately. Figure 1 displays examples of TLS and their location. The interobserver agreement ranged between 0.15 and 0.56 for non-dichotomized data, and 0.03–0.45 for dichotomized data. Only the non-dichotomized data for the assessment of tumor TLS displayed a moderate interrater agreement (ICC = 0.56, CI: 0.50–0.62, *P* <.001). The deviating cases were re-evaluated, and all subsequent statistical analyses were based on the specialist’s annotation after review by a pulmonary pathologist (PM).

**Table 1:**
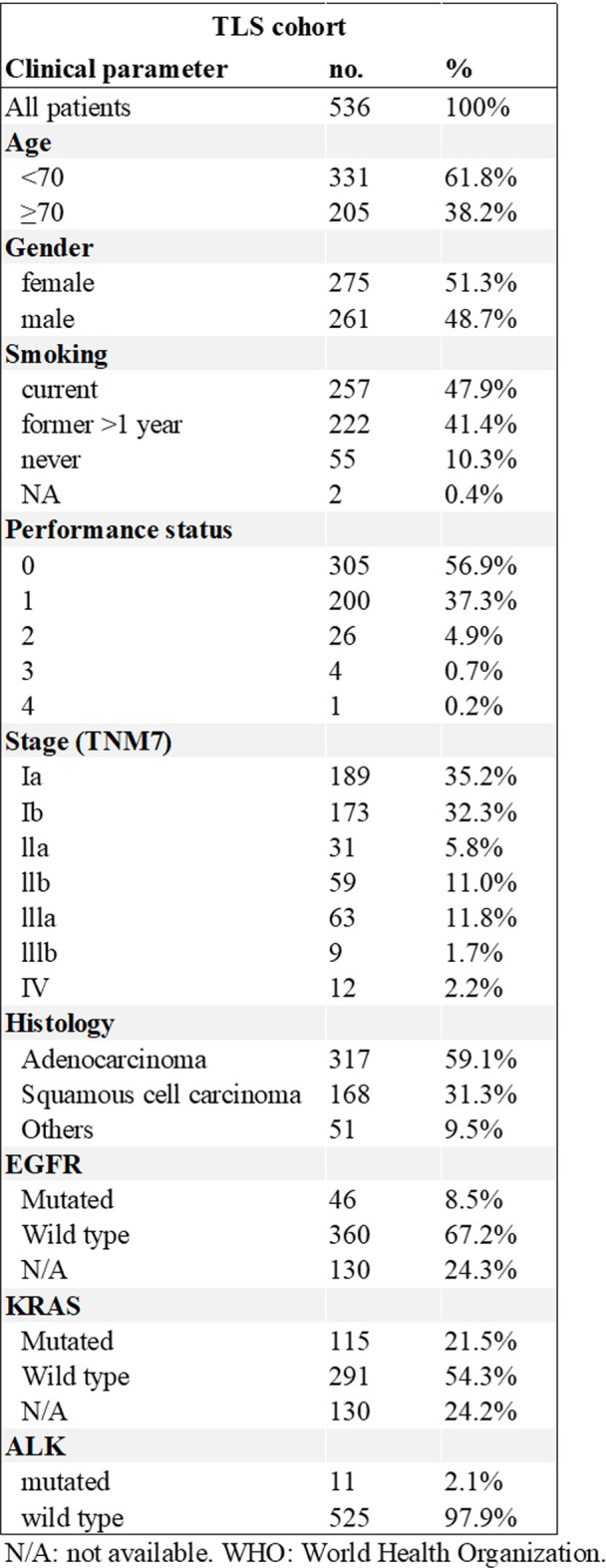
Clinical characteristics of patients included in the TLS cohort

### TLS are a common phenomenon in NSCLC with higher abundance in squamous cell cancer

**Fig. 2:**
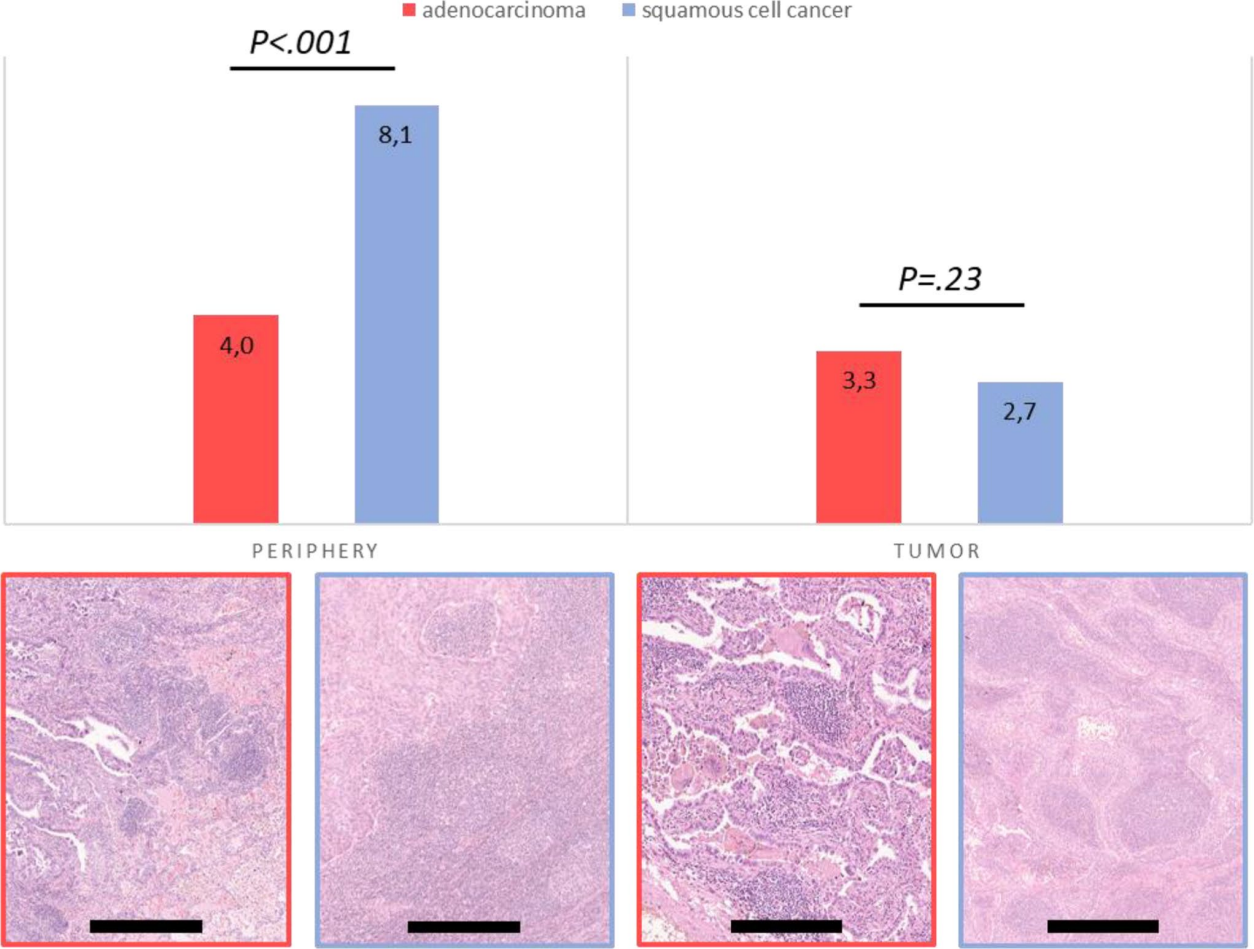
Histogram displaying the difference in mean number of TLS in the periphery and tumor compartment between adenocarcinomas and squamous cell cancers, together with representative histological photographs. Scale bar indicate 100 μm

The study cohort included 317 (59%) adenocarcinomas, 168 (31%) squamous cell cancers, and 51 cases (10%) of other histologies (e.g. large-cell neuroendocrine carcinoma, large cell carcinoma, adenosquamous carcinoma). TLS were present in 86% of the tumors, showing one or more TLS, whereas in 14% of cases, not a single TLS was identified. The mean number of TLS were 8 TLS/slide and case, calculated from all cases with and without TLS. Of the total 4852 TLS, 314 TLS (7%) showed germinal centers and were regarded as mature TLS. TLS were more abundant in squamous cell cancer (mean 10.8 TLS/case) than in adenocarcinoma (mean 7.3 TLS/case; *P* <.001) (Fig. 2) with a significantly higher number in the periphery. Peripheral TLS were more abundant in smokers in the total cohort (adj. *P* =.04) and in the adenocarcinoma subgroup (adj. *P* =.04). The other clinical parameters (age, stage, and performance status) were not associated with the presence of total TLS, TLS in the periphery, within the tumor area, or mature TLS (all comparisons *P* >.05) (Suppl. Table 2A-C).

### TLS were overall not associated with the mutational background of cancer

Information on gene expression data for 82 different lung cancer-related genes, including *EGFR*, *KRAS*, *ALK*, *PIK3CA*, *STK11*, and *TP53*, were available for 165 included patients.

The presence of specific tumor driver genes (*KRAS*, *ALK*, and *EGFR*) or suppressor genes (*STK11* and *TP53*) was analyzed in relation to the abundance of TLS (Suppl. Table 3). Tumors harboring mutations in *KRAS* (*P* =.003) and *STK11* (*P* =.03) did overall present with less TLS. However, only the negative association with *KRAS* remained significant after adjustment for multiple testing (*P* =.02). Because *KRAS* is generally only present in adenocarcinomas, this relation is likely driven by histology. When adenocarcinomas were analyzed separately, the *KRAS* and TLS association receded. Cases with high TMB had higher numbers of peripheral TLS (*P* =.03), however no association was seen for tumor TLS (*P* =.19) (Suppl. Figure 1).

Taken together, there was no clear statistical evidence that driver mutations or suppressor genes were connected to the presence of TLS. The significant association of peripheral TLS with the TMB deserves further validation.

### TLS were associated with overall survival

**Fig. 3:**
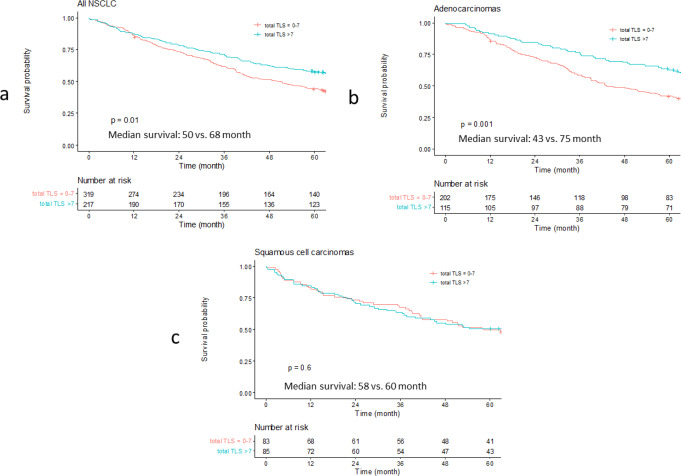
Kaplan-Meier curves display the impact of survival with regard to the presence of TLS in the tissue sections. **A**: Kaplan-Meier curve for all NSCLC stratified by the total amount of TLS (0–7 vs. >7). **B**: Kaplan-Meier curve for adenocarcinomas stratified by the total amount of TLS (0–7 vs. >7). **C**: Kaplan-Meier curve for squamous cell cancers stratified by the total amount of TLS (0–7 vs. >7). *P*-values were based on the log-rank test and present the comparison of both groups

Information on clinicopathological parameters (age at diagnosis, tumor stage, performance status, smoking) and overall survival was available for all 536 included patients. The presence and distribution of TLS were analyzed regarding their prognostic impact. The Kaplan-Meier curve indicated that the presence of TLS (cut-off > 7 TLS per slide) was correlated with longer overall survival (*P* =.01). This association was mainly driven by the location of TLS in the tumor periphery in the total cohort (*P* =.004) and in the adenocarcinoma subset (*P* =.001) (Fig. 3). These specific associations (peripheral TLS and in adenocarcinoma) were also retained when the Uppsala I and II cohort were analyzed separately (Suppl. Figures 2–3).

The presence of TLS with germinal centers showed a similar positive impact on survival as the total TLS count. However, this association was mainly driven by squamous cell carcinomas cohort (*P* =.02), and not by the adenocarcinoma subgroup (*P* =.44) (Suppl. Figure 4).

**Fig. 4:**
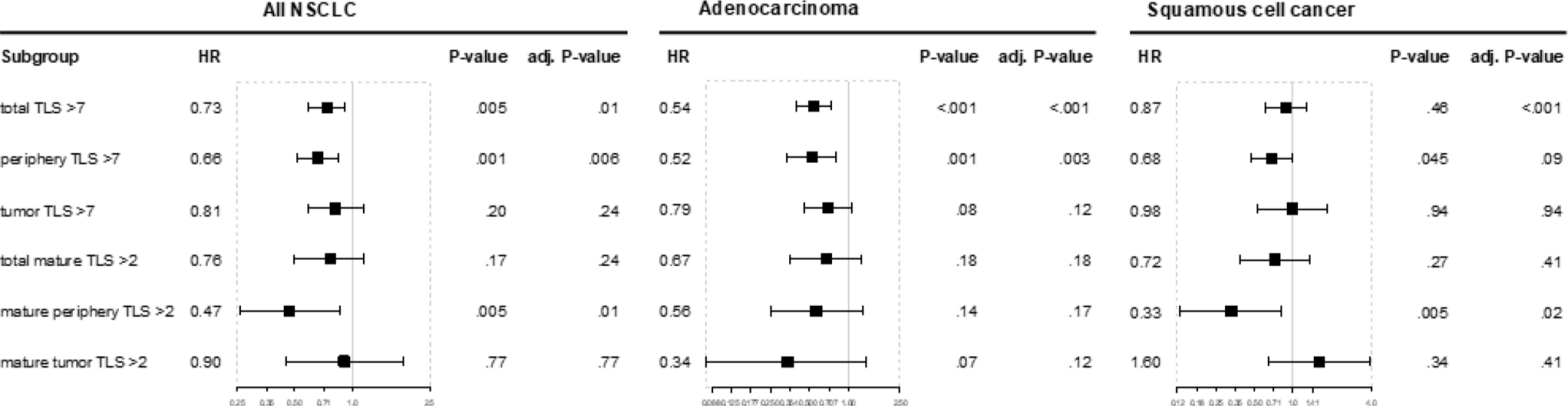
Forest plots display the association of TLS with overall survival. A Cox regression model was applied to analyze the association of the presence of TLS in all NSCLC, adenocarcinoma and squamous cell cancer, respectively. The Cox regression model was controlled for stage, age, smoking and performance status (*P*-value). *P*-values were also adjusted for multiple testing (adj. *P*-value)

The observed favorable prognostic impact was independent of other clinical parameters (stage, age, performance status, and smoking history) in the multivariate Cox-regression model (Table 2), as illustrated in the forest plots (Fig. 4) (Suppl. Table 4). Higher total TLS and higher peripheral TLS with or without germinal centers were associated with better survival in the univariate and multivariate Cox regression model, whereas mature TLS in the tumor area were not.

**Table 2:**
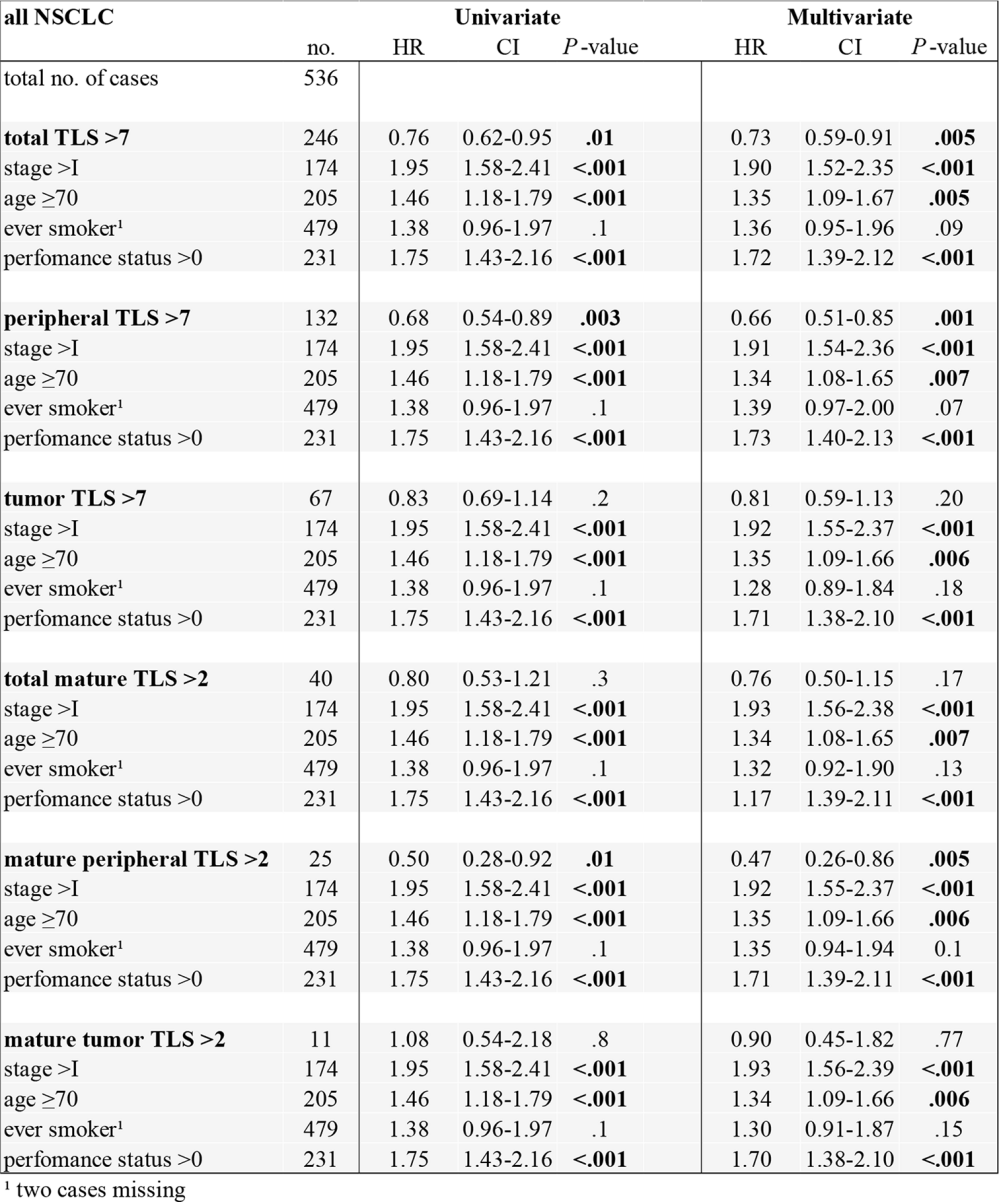
Univariate and multivariate Cox regression for TLS in our NSCLC cohorts. The multivariate regression model for the numbers of TLS (dichotomized 0–7 vs. >7) was controlled for the clinical parameters age (< 70 vs. ≥70), stage (I vs. II-IV), smoking (never vs. ever smoker), and performance status (0 vs.1–4)

TLS: tertiary lymphoid structure; NSCLC: non-small cell lung cancer; HR: hazard ratio; CI: confidence interval

In conclusion, we confirmed that TLS have a favorable prognostic impact on survival, with peripheral TLS likely to be more important and with a dominant survival association in adenocarcinoma cases. It must be stressed that these analyses should be interpreted with caution because of cut-off optimization, multiple testing, and subgroup analysis with different statistical power.

### Correlation with immune cell infiltrates

Information on immune cell infiltrates, including lymphocytic markers (CD3, CD4, CD8, CD20, FOXP3), anti-inflammatory macrophages/myeloid cells (CD163), plasma cells (CD138) and the checkpoint molecule PD-1 and well as PD-L1, was available for 271 patients. This data was obtained from TMAs containing central areas of the tumor tissue and microscopic estimations of immunohistochemically stained slides.

Associations between the location and presence of TLS to compartment specific infiltration of immune cells or marker expression, revealed several significant associations even after adjustment for multiple testing (Suppl. Table 5). The strongest associations were observed when the TLS in the tumor periphery were considered: Higher number of peripheral TLS were associated with higher numbers of stromal CD4+, CD8+, CD20+, FOXP3+, CD138+, and PDL1 + cells, as well as the infiltration of CD3+, CD4+, CD8+, CD20+, FOXP3+, PD1+, and PD-L1 + cells in the tumor cell compartment. Some associations were stronger when only mature peripheral TLS were associated with infiltrates. Only one negative association was observed: When TLS were located within the tumor, this region comprised fewer CD163 + cells (considered as M2-like macrophages). Taken together, we observed a strong association of TLS to specific immune cell subsets. Again, results should be interpreted with caution, since an inflamed phenotype with a generally high immune cell infiltration might be responsible for these seemingly specific associations.

## Discussion

This study describes the spatial abundance of TLS in a large, extensively characterized cohort of resected NSCLC patients. We confirmed the favorable prognostic impact of TLS counts, independent of stage, age, smoking, and performance status. The favorable survival association depended on the TLS location in the tumor periphery and was more dominant in the adenocarcinoma subtype. The mutation status did not impact the presence of TLS, but overall mutational load might be of relevance. Not surprisingly, TLS correlated with a generally higher infiltration of immune cells, particularly the lymphocytic subsets, including T-regulatory cells.

To our knowledge, this is one of the most extensive and integrative studies evaluating TLS formation in NSCLC. Some previous studies (Suppl. Table 6) have addressed the clinical impact of TLS in lung cancer. These efforts have primarily relied on the aid of immunohistochemical stainings for an accurate identification of TLS. In the seminal study of Dieu-Nosjean and coworkers, the marker DC-LAMP was used to identify TLS in a cohort of 74 NSCLC tumors. They demonstrated an association with overall survival, disease-specific survival, and disease-free survival in an univariate analysis [27]. The same group confirmed the favorable impact of DC-LAMP aggregates in a cohort of 386 operated NSCLC patients in the univariate analysis, however not in a multivariate analysis [32].

One of the most extensive studies, including two Norwegian cohorts with in total 553 NSCLC patients, used a simple double staining (pan-cytokeratin and CD8) to highlight TLS for quantification. They demonstrated that all obtained metrics, the total amount, the compartment-specific amount, and the amount of germinal center positive TLS, were associated with survival in univariate and multivariate analysis in histological subtypes and within different stages [39].

Only a few studies used traditional quantification on H&E slides. Silina and coworkers evaluated TLS visually in a cohort of 138 squamous cell cancer patients. They also showed a substantial survival benefit for patients with high TLS in the univariate and multivariate survival analysis [38]. The study of Tamiya focused on stage 1B adenocarcinoma and quantified only TLS with germinal centers, again with independent favorable prognostic impact [28].

Since most TLS metrics and quantification methods provided comparable prognostic information, we applied the most straightforward approach; basic visual assessment of H&E slides which can be performed directly on the diagnostic slide by a trained pathologist, and can readily offer additional prognostic risk stratification after surgical resection.

While several factors limit the certainty of our findings, including cut-off optimization, multiple testing, the exclusive association in adenocarcinoma, and moderate interrater agreement, we believe our results underscore the diagnostic potential of the routine TLS assessment on H&E sections. For integration into the clinical workflow, specific training and clearer guidelines will be essential to improving interobserver agreement. Refining TLS definitions on H&E sections, developing reproducible criteria for slide selection and area-of-interest identification, and implementing standardized counting procedures, as well as and training programs, will likely further increase the prognostic value of TLS counts. For some patients, this added prognostic information might be decisive in guiding adjuvant treatment after surgical resection.

Our study showed that the occurrence of TLS is not an independent phenomenon but is strongly linked to the infiltration of other immune cells. For the immune cell scores we used TMAs that were previously annotated by a pathologist based on immunohistochemical stainings and microscopic estimations [3]. We and others have shown, that TMAs relatively well represent the general immune phenotypes of the tumor [8, 49–52]. It should be noted that the cores only display representative central tumor areas and were not selected to include specifically TLS areas, thus the immune cell profiles do not describe the composition of the TLS, but an association with the general tumor immune phenotypes.

The direct infiltration of CD8 + effector cells in the tumor cell islands indicates a biological effect of TLS, which is accompanied by inhibitory signals with upregulation of PD-L1 on tumor cells and PD1 on lymphocytes. This phenomenon is recognized as an immune exhaustion phenotype [53, 54]. In parallel with higher TLS and CD8 + counts, the infiltration of regulatory T-cells and “inhibitory” M2-line macrophages increases. This finding is in line with the study of Rakaee [39], where the presence of TLS was associated with an immune-exhausted gene expression signature.

This immune cell-rich cellular composition presents the typical inflamed immune phenotype that is constantly associated with a better prognosis [14, 55]. Therefore, it can be argued that the development of TLS and the inflamed immune microenvironment results from a generally active immune system, and is not necessarily an indication of a specific anti-cancer response. TLS might also be the effect of inflammation or infection, e.g., through obstructive changes during tumorigenesis.

Our study though, supports the concept that TLS are involved in a specific anti-cancer immune response, based on the relation between TLS and the TMB, as a surrogate of neoantigen expression with mutated peptides causing specific activation of T- and B-lymphocytes [56, 57].

However, as the previous studies, our study was also retrospective and descriptive in nature, thus it is challenging to interpret associations. The weakness of cut-off optimization and multiple-testing may lead to an overestimation of associations. Therefore, an independent study using a more rigorous statistical approach with a training and validation cohort is warranted to obtain the ultimate evidence about the prognostic impact on TLS metrics on HE-sections. Also the addition of progression-free survival would be of benefit.

Furthermore, a direct comparison of TLS analysis on HE sections with matched immunohistochemical stained slides would help to estimate the value of one strategy over the other. Functional studies are warranted to understand the mechanism of how TLS develops and which immune reactions TLS mediate. In an elegant approximation, the group of Germain and colleagues showed that plasma cells isolated from the local tumor environment produced IgG and IgA antibodies against classical cancer testis antigens, and that the presence of antibodies was associated with densities of follicular B-cells [31], giving support that tumor-specific plasma cells develop in local TLS.

As a final consideration, we probably did not evaluate the impact of TLS in the most clinically relevant context. The NSCLC patients analyzed in this study were all surgical treated, but were otherwise heterogeneous, diagnosed in a time frame of 15 years. Treatment has changed, and different adjuvant chemotherapy regimens were introduced that may directly impact survival [58]. This becomes even more relevant when immunotherapy (alone or in combination) will be used as main neoadjuvant or adjuvant option [59, 60]. It will be intriguing to evaluate the impact of TLS in this context. One preliminary study [61] demonstrated a strong association between TLS maturation and pathological response in the resected tumor specimens after neoadjuvant immunochemotherapy. In this study, TLS were also predictive for disease-free survival.

The era of digital pathology will further advance the evaluation of tissue patterns for diagnostic purposes. Automated whole slide scannings and artificial intelligence-based learning algorithms will reduce interobserver variability and increase the accuracy of scoring. One study compared TLS detection using deep convolutional neural networks and found a 93% specificity at 95% sensitivity of the image analysis approach compared to annotations of a histopathologist [62]. Some recent studies applied such TLS recognition algorithms already successfully to predict survival [63–65].

Digital evaluation might not only include not only TLS, but also other tumor or stroma features related to survival and therapy benefit. This information should be integrated together with clinical and molecular data to further enhance prognostic and predictive performance [66, 67]. As a computational tool, it could be easily integrated into the standard evaluation of digital H&E slides, either assisted or fully automated.

In conclusion, our study demonstrated that the enumeration of TLS in diagnostic H&E sections provides comparable clinical prognostic information as previously described immunohistochemistry-based studies. The observed associations with anti-tumorigenic immune cell infiltrates and regulatory cell elements support the assumption that TLS are of biological relevance. A simple TLS quantification deserves further support as a routine metric in clinical pathology reports of NSCLC patients.

## Electronic supplementary material

Below is the link to the electronic supplementary material.

Supplementary Material 1

Supplementary Material 2

## Data Availability

No datasets were generated or analysed during the current study.
