# Supplementary material for: Spatial distribution of tertiary lymphoid structures in the molecular and clinical context of non-small cell lung cancer
Source: Cell Oncol (Dordr). 2025 Mar 3;48(3):801–13. doi: 10.1007/s13402-025-01052-x (PMC12119696; doi:10.1007/s13402-025-01052-x)

Spatial distribution of tertiary lymphoid structures in the molecular and clinical context of non-small cell lung cancer.
Cellular Oncology

Hedvig Elfving[1], Hui Yu, Kaleab Kassete Fessehatsion, Hans Brunnström, Johan Botling, Miklos Gulyas, Max Backman, Amanda Lindberg, Carina Strell, Patrick Micke
[1]Department of Immunology, Genetics, and Pathology, Uppsala University, Uppsala, Sweden. hedvig.elfving@igp.uu.se

Supplemental figures

Suppl. Figure 1. Box plot for the correlation between tumor mutational burden (TMB) and number of peripheral TLS (a) and tumor TLS (b).

Suppl. Figure 2. Uppsala I cohort and Kaplan-Meier curves for the association of TLS with survival, for the whole cohort (all NSCLC) (n=272), and the histological subgroups adenocarcinoma (n=154) and squamous cell carcinoma (n=86). The total TLS counts are dichotomized 0-7 vs. >7, mature TLS are dichotomized 0-2 vs. >2.

Suppl. Figure 3. Uppsala II cohort and Kaplan-Meier curves for the association of TLS with survival, for the whole cohort (all NSCLC) (n=264), and the histological subgroups adenocarcinoma (n=163) and squamous cell carcinoma (n=82). The total TLS counts are dichotomized 0-7 vs. >7, mature TLS are dichotomized 0-2 vs. >2.

Suppl. Figure 4. Kaplan-Meier curves for the association of TLS with survival, for the whole cohort (all NSCLC) (n=536), and the histological subgroups adenocarcinoma (n=317) and squamous cell carcinoma (n=168). The total TLS counts are dichotomized 0-7 vs. >7, mature TLS are dichotomized 0-2 vs. >2.

Suppl. Figure 1

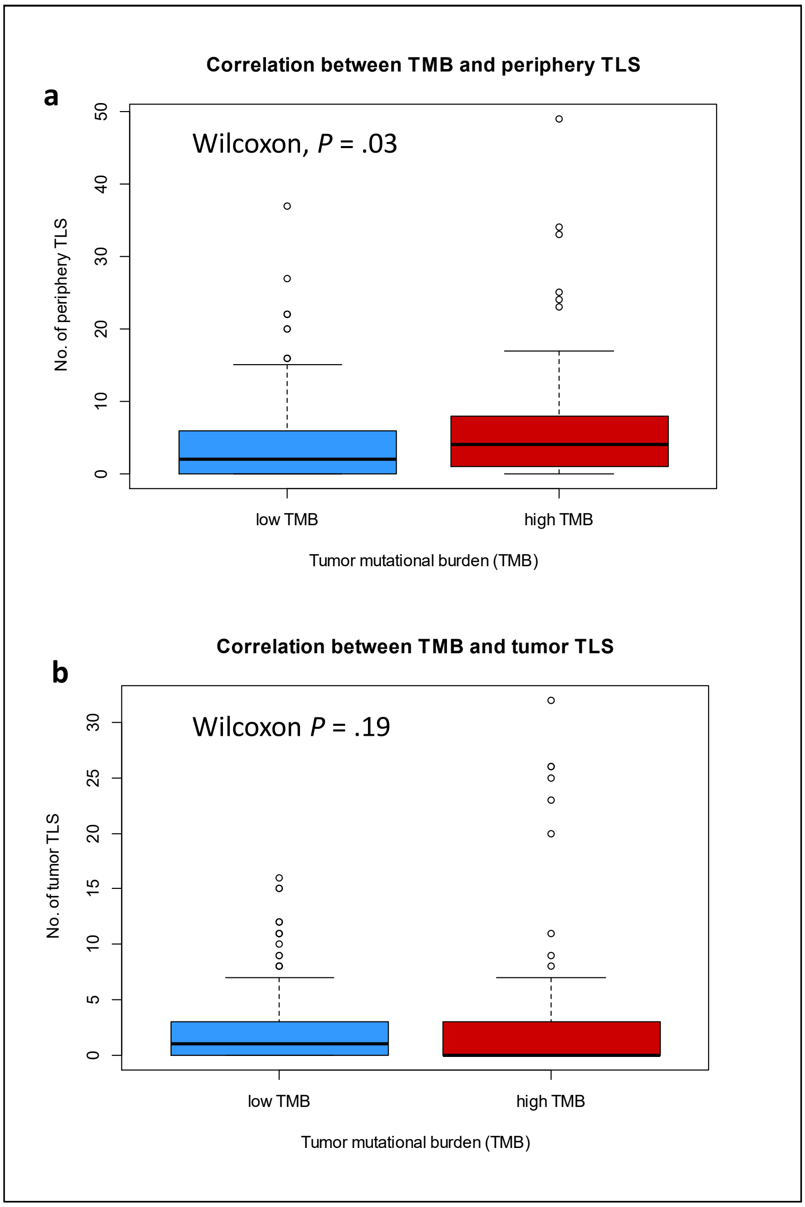

Suppl. Figure 2

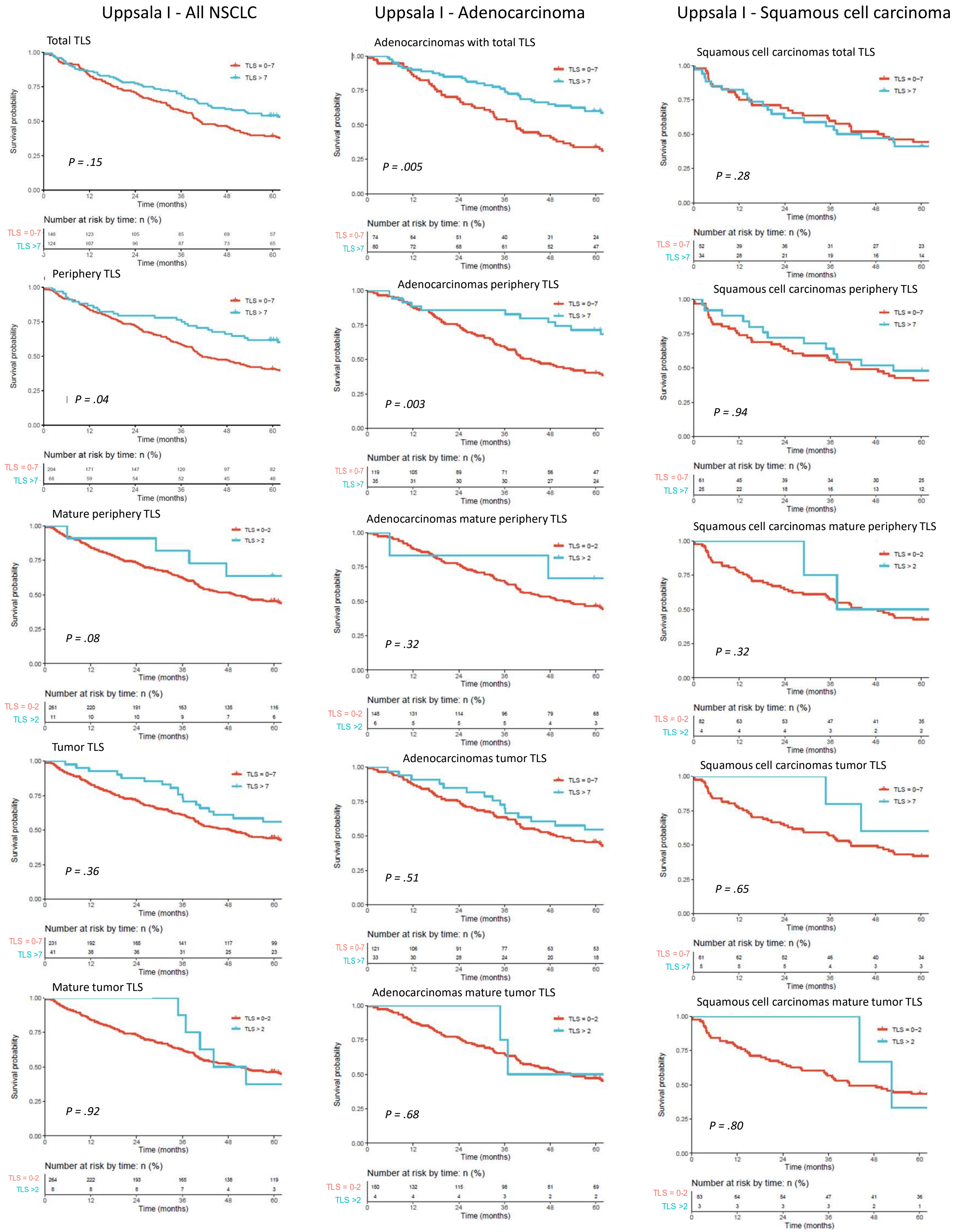

Suppl. Figure 3

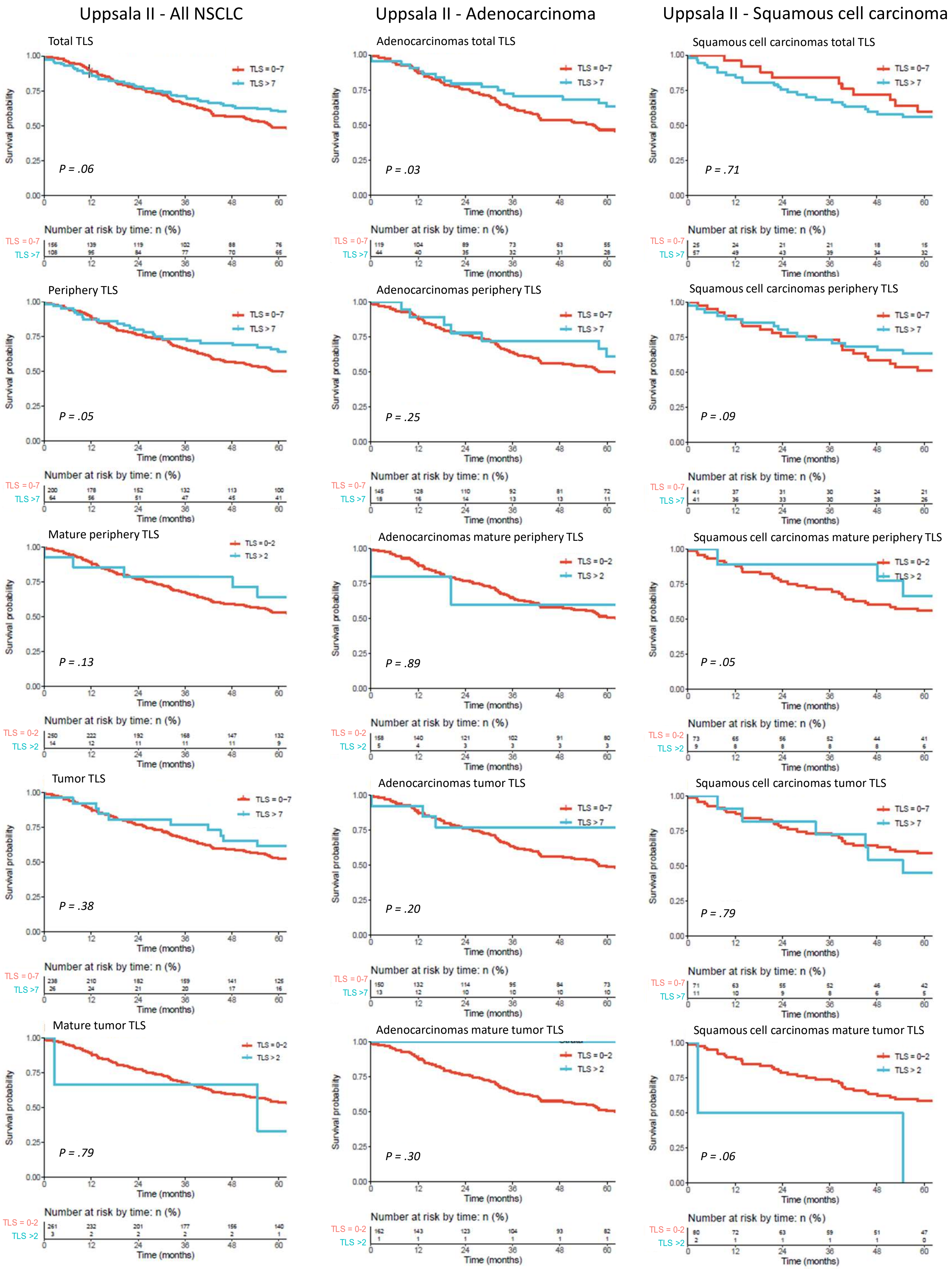

Suppl. Figure 4

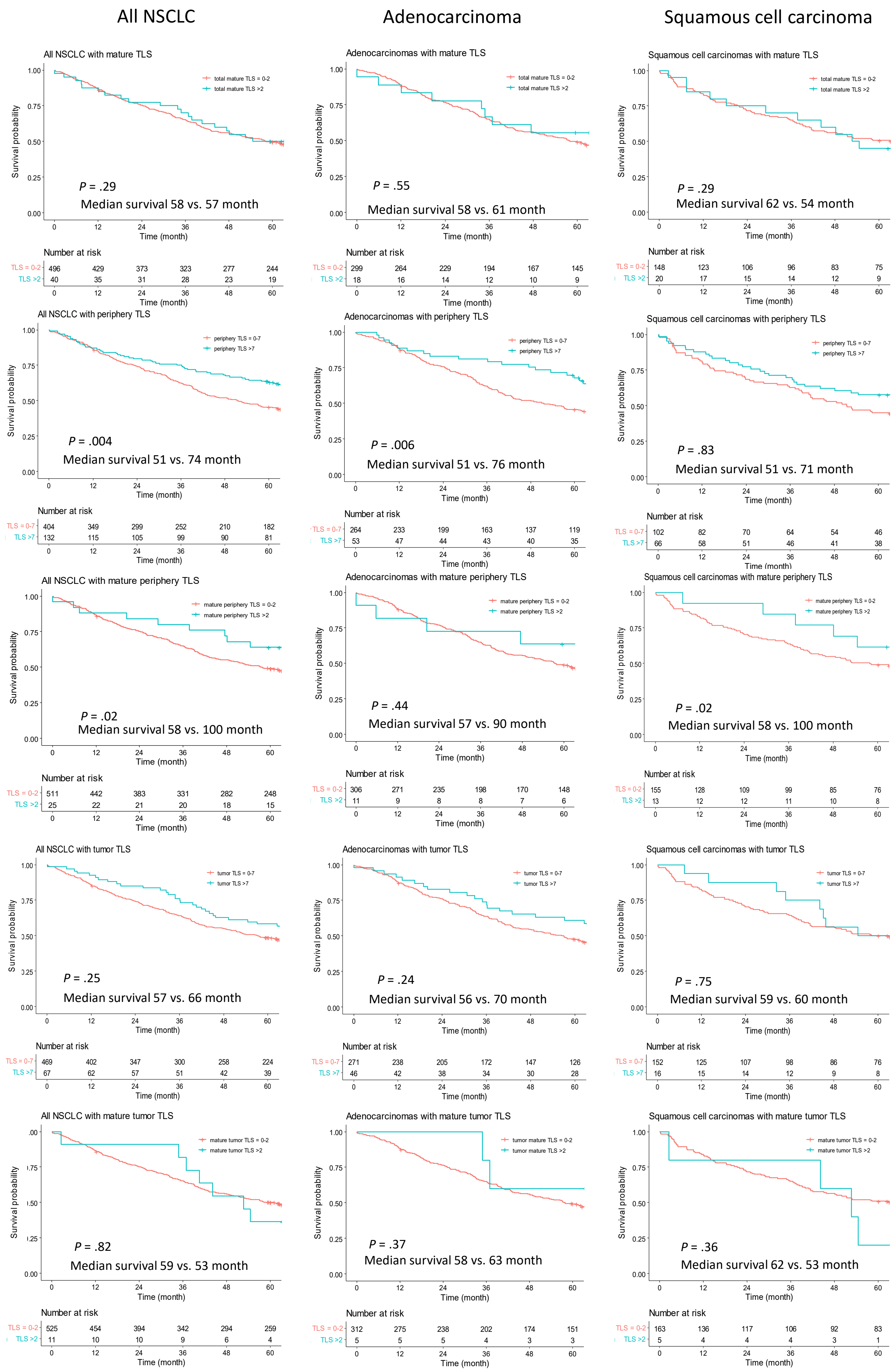

Supplement: Supplementary file 1 — Supplementary Material 1 [file 13402_2025_1052_MOESM1_ESM.pdf]
